# Specific patterns of gene space organisation revealed in wheat by using the combination of barley and wheat genomic resources

**DOI:** 10.1186/1471-2164-11-714

**Published:** 2010-12-19

**Authors:** Camille Rustenholz, Pete E Hedley, Jenny Morris, Frédéric Choulet, Catherine Feuillet, Robbie Waugh, Etienne Paux

**Affiliations:** 1 INRA UMR 1095, Génétique Diversité et Ecophysiologie des Céréales, 63100 Clermont-Ferrand, France; 2 Scottish Crop Research Institute, Invergowrie, Dundee, DD2 5DA, UK

## Abstract

**Background:**

Because of its size, allohexaploid nature and high repeat content, the wheat genome has always been perceived as too complex for efficient molecular studies. We recently constructed the first physical map of a wheat chromosome (3B). However gene mapping is still laborious in wheat because of high redundancy between the three homoeologous genomes. In contrast, in the closely related diploid species, barley, numerous gene-based markers have been developed. This study aims at combining the unique genomic resources developed in wheat and barley to decipher the organisation of gene space on wheat chromosome 3B.

**Results:**

Three dimensional pools of the minimal tiling path of wheat chromosome 3B physical map were hybridised to a barley Agilent 15K expression microarray. This led to the fine mapping of 738 barley orthologous genes on wheat chromosome 3B. In addition, comparative analyses revealed that 68% of the genes identified were syntenic between the wheat chromosome 3B and barley chromosome 3 H and 59% between wheat chromosome 3B and rice chromosome 1, together with some wheat-specific rearrangements. Finally, it indicated an increasing gradient of gene density from the centromere to the telomeres positively correlated with the number of genes clustered in islands on wheat chromosome 3B.

**Conclusion:**

Our study shows that novel structural genomics resources now available in wheat and barley can be combined efficiently to overcome specific problems of genetic anchoring of physical contigs in wheat and to perform high-resolution comparative analyses with rice for deciphering the organisation of the wheat gene space.

## Background

The term "gene space" refers to the fraction of the genome corresponding to protein coding genes and, by extension, to the distribution of these genes [1]. In large genomes that contain abundant repetitive DNA, it encompasses also the notion of regions containing genes, the so-called gene-rich regions, surrounded by gene-poor regions composed of repeats [2].

With the growing number of sequenced plant genomes, it becomes obvious that the distribution pattern of genes is far from random and not universal across the plant kingdom. Small plant genomes, such as *Arabidopsis thaliana *(125 Mb), *Brachypodium distachyon *(272 Mb) and *Oryza sativa *(389 Mb) exhibit fairly homogenous gene distribution along their chromosomes [3-5]. The transition from a homogenous to a non-homogenous gene distribution seems correlated to the genome size. Indeed, in intermediate size genome, such as *Populus trichocarpa *(485 Mb) and *Vitis vinifera *(487 Mb), large regions alternating between high and low gene density were observed [6,7], whereas larger genomes, such as *Glycine max *(1115 Mb) and *Zea mays *(2300 Mb), display an increasing gradient of gene density from the centromere to the telomeres [8,9].

Because of its size (17000 Mb), allohexaploid nature (A, B and D-genomes) and high repeat content (>80%) [10], the bread wheat (*Triticum aestivum *L.) genome is among the largest and most complex plant genomes and has always been considered too complex for molecular analyses. As a result, no genome sequence is available yet and very little is known about the organisation of the wheat gene space. The first insights were obtained from the mapping of wheat gene-based markers in wheat aneuploid genotypes called deletion lines where fragments of chromosomes or deletion bins are missing [11]. Based on EST and *Pst1 *genomic clone mapping, Erayman *et al*. [12] suggested a very heterogeneous distribution of the genes along the wheat chromosomes, with 94% of the genes being located in only 29% of the entire wheat chromosomes and mostly at their telomeric parts. In contrast, by EST mapping on chromosome group 3 deletion bins, Munkvold *et al*. [13] observed a slight gradient of the gene density along the chromosomes as well as a significant number of genes in the most proximal bins thereby suggesting a more homogeneous distribution. More recently, individual BAC sequencing [14,15] confirmed a rather homogeneous gene distribution in wheat with an average of one gene per BAC. Finally, Choulet *et al*. [16] investigated megabase-sized regions from various parts of chromosome 3B and indicated that the gene-free regions are much smaller than expected by Erayman *et al*. [12], *i.e*. not larger than 1 Mb. Moreover, they found evidence for a slight gradient (twofold) of the gene density distribution from the centromere to the telomeres. Thus, additional whole genome or whole chromosome analyses are needed to better characterize the gene space organisation in wheat.

We recently constructed a physical map of chromosome 3B, the largest wheat chromosome (1 Gb, 2.5 times the whole rice genome) [17]. The map consists of 1036 contigs spanning 811 Mb, of which 611 Mb are anchored with 1443 molecular markers. However, very few contigs are anchored by gene-derived markers. Indeed despite the development of genomic resources, such as extensive marker collections and saturated genetic maps [18-20], genetic mapping of genes in wheat is still hampered by the lack of polymorphism and the presence of the three homoeologous copies of each gene. As a result, no high density transcript genetic map is available. In contrast, several gene maps have been constructed for barley [21-25] (*Hordeum vulgare *L.) that diverged from wheat ~10-12 MYA [26,27] and belongs to the same tribe (Triticeae). With a size of 4.9 Gb [10] and a repeat content of over 80% [28], the diploid barley genome (2n = 14) is very similar to the wheat subgenomes and several mapping studies have demonstrated a high collinearity between barley and wheat [26,27,29-32].

Here, we wanted to explore the possibility of using barley transcript genetic maps as a surrogate to anchor and order the wheat physical contigs. BAC pools representing the minimal tiling path (MTP) of wheat chromosome 3B were hybridised onto barley expression microarrays to identify the location of genes along the wheat 3B physical map. The results show that such barley-wheat cross-hybridisations represent high-throughput cost-efficient approaches for anchoring genes on wheat physical maps and for performing comparative genomics studies between wheat and other grass genomes. In addition, the possibility to locate genes precisely within BAC contigs that were anchored by other markers onto the chromosome 3B enabled us to gain new insights into the distribution of genes along a wheat chromosome.

## Results and discussion

### A high throughput anchoring method

To assess the efficiency of wheat-barley cross-species hybridisation for gene-based physical map anchoring, a barley Agilent 15K unigene microarray was hybridised with 60 three-dimensional (plate, row, column) BAC pools from the MTP of the wheat chromosome 3B [17]. After signal quantification and normalisation, hybridisation data were evaluated with four complementary scoring methods to reliably locate as many barley gene homologs as possible on the wheat BACs (see Methods). Using the most stringent "automated scoring" method, 3355, 3401 and 3286 probes were identified as positive with the plate, row and column pools, respectively. Deconvolution of the pool data led to the identification of 571 unambiguous BAC addresses for 566 unigenes, defining 561 unique genomic loci and 5 duplicated loci. The less stringent "boxplot scoring" method led to the identification of 6205, 5103 and 6761 positive probes for the plate, row and column pools, respectively. With this method, 770 probes having unambiguous BAC addresses were identified, including 481 that were already identified with the "automated" method. Out of the 289 newly identified probes, we selected 86 probes (100 loci) that correspond to the most robust data (*i.e*. located on two to three overlapping BACs). Finally the "semi-automated" and the "manual scoring" methods added additional BAC addresses for 13 and 78 probes respectively that showed missing coordinates with the two other methods due to technical limitations (detailed below).

In total, the combination of four methods enabled us to identify 762 unambiguous wheat BAC addresses for 743 barley probes. A BLASTN search [33] against the Triticeae repeat database TREP [34], indicated that five probes had high sequence identity (>86%) with TEs and were removed from further analysis. Each of the remaining 738 non TE-related genes was assigned to one to three wheat BACs resulting in 757 gene loci identified on the wheat chromosome 3B physical map [17]. These barley unigenes were located on 624 wheat BACs that corresponded to 388 individual contigs of 187 kb to 3.8 Mb and 86 singletons.

We tested the reliability of the 757 genes using the sequenced contigs available on chromosome 3B [16]. We found that 74% (23/31) of the genes located on the sequenced contigs through hybridisation gave a hit on the sequenced contigs after a BLASTN analysis. Out of these genes, 91% (21/23) matched a gene on the sequenced contigs at their expected location. Eight unigenes (26%) were assigned to these contigs but their position was not supported by sequence information. Several hypotheses can be proposed to explain such discrepancy between hybridization and sequencing data, including false positives, misassembled MTP BACs or gaps in the sequence. In addition, 30 out of the 15208 barley Agilent microarray unigenes matched a gene after a BLASTN analysis against the sequenced contigs but were not located on a BAC through hybridisation. However the sequence identity of these 30 unigenes (84%) was lower than the sequence identity of the 21 unigenes located on the sequenced contigs through hybridisation (90%). These unigenes would therefore hardly be located on a BAC through hybridisation. These data validated this cross-species hybridisation approach as a powerful and reliable method to map genes to BAC contigs.

The 738 probes correspond to roughly 40% of the barley unigenes that were expected to be present on the wheat chromosome 3B physical map. Indeed, chromosome 3 H accounts for approximately 14.8% of the barley genome [35,36]. Assuming a comparable gene density for all barley chromosomes, 2250 probes out of the 15208 unigenes are expected to be located on chromosome 3 H. As the MTP covers 82% of the whole wheat chromosome 3B, about 1845 probes should in theory be present on the wheat chromosome 3B physical map assuming that all barley genes are conserved in wheat. The difference of 60% between expected and observed results could be explained by both biological and technical limitations of our experiment. First, sequence divergence between wheat and barley genes may have significantly impacted the efficiency of this approach. Letowski *et al*. [37] estimated that hybridising a probe and a DNA target sharing 90% of sequence identity results in 73% to 99% decrease in hybridisation signal intensity compared to a probe and a DNA sharing 100% of sequence identity. Blasting the barley 60-mer probes against the 6162 wheat *cv*. Chinese Spring full-length cDNA dataset [38] revealed that 56% of the hits show more than 10% nucleotide divergence (86% identity on average). Moreover we found that the unigenes located on the sequenced contigs of the wheat chromosome 3B [16] through BLASTN and hybridisation showed a significantly higher sequence identity (90%) compared to the ones that were located on the sequenced contigs through BLASTN only (83%) (T-test, *P-*value = 5E-6). Therefore, one can estimate that sequence conservation played a key role in the detection of hybridisation signals and that more than half of the potentially positive barley probes generated a near undetectable hybridisation signal with the wheat BACs. A second origin of the discrepancy likely originates from the presence of gene families located at multiple loci. The wheat genome is allohexaploid (three subgenomes: A, B and D) and at least one copy of each wheat gene is expected to be present on the three homoeologous chromosomes. In addition, there is increasing evidence for high level of tandem and interchromosomal duplication events in wheat and perhaps barley genomes since their divergence from the other grasses [16,39]. Thus there is a good probability that some genes are found in multiple copies on chromosome 3B. Such genes can result in multiple non-overlapping BAC addresses that cannot be resolved without ambiguity and are therefore excluded from our analysis. Another critical point affecting the efficiency of the approach lies in the putative heterogeneity of the BAC pools. Indeed, each three-dimensional MTP pool contains more than 300 BACs (see Methods), making it difficult to guarantee equimolarity for all BACs. In some extreme cases, this heterogeneity in individual BAC quantity may lead to weak signal intensity for positive probes resulting in missing coordinates. These two limitations could be circumvented by the use of six-dimension pools of the complete chromosome 3B BAC library [40]. However, such pools would have required almost 3 times more hybridisations than the three-dimensional MTP pools (177 vs. 60) thereby reducing the cost-efficiency of the approach.

Despite these limitations, this single experiment permitted the localisation of 738 genes on the wheat chromosome 3B contigs and allowed us to get novel information for the order of the BAC contigs along the chromosome based on the barley EST genetic maps. So far, genetic mapping of genes in wheat has been hampered by the lack of polymorphism in the genic sequences and the presence of several homoeologous copies. As a consequence, only a third of the 680 markers located on the chromosome 3B genetic map constructed using the 'neighbours' approach correspond to ESTs [17]. Here, we established a barley whole genome neighbour map using the same criteria as the IBM neighbour map of maize [41] and used it to assess the order of wheat contigs based on the EST order found on the genetic map. Out of the 738 probes assigned to BAC contigs of wheat chromosome 3B, 308 (42%) were mapped to the barley neighbour map including 209 on chromosome 3 H and 99 on other chromosomes. Using the barley 3 H mapping data, 151 BAC contigs and 20 singletons from the wheat chromosome 3B physical map were genetically ordered. Only 30% of these contigs were previously ordered genetically using the wheat chromosome 3B neighbour map, whereas 44% were mapped to the wheat chromosome 3B deletion bin map, but not ordered in bins, and 26% were not anchored at all [17]. In addition, it is worth noting that 36% of the 151 contigs are only anchored by gene-based markers. This is consistent with the results of Paux *et al*. [17] who showed that some regions of the genome can only be anchored by specific types of markers (ESTs, SSRs, ISBPs) and that 35% of the contigs were anchored by ESTs only. Therefore, we conclude that the cross-hybridisations of wheat BAC pools with barley expression microarrays is a straight forward approach to order wheat contigs with gene-based markers without the difficulty of EST genetic mapping in wheat.

Moreover, the total cost for these 60 pool hybridisations on 15 microarrays was approximately 8800 USD. For the same price, PCR screening of individual EST markers on the same BAC pools (including primers and amplification) would only have allowed testing of 500 markers. Thus, the method is a cost-efficient alternative to PCR-based physical map anchoring. However, despite its convenience and its cost-efficiency, this technique is still limited in the number of contigs anchored and ordered but it would be greatly improved by technological developments in the near future. First, use of the barley 44K Agilent expression microarray will significantly increase the number of positive probes, regardless of the experiment efficiency. Second, as large amounts of barley SNPs are becoming available [22], the number of genetically mapped genes will increase in the coming years, therefore improving the efficiency of the anchoring strategy.

Finally, wheat-barley cross-species hybridisation is a convenient, cost-efficient and relatively high-throughput approach for gene-based physical map anchoring and ordering of wheat BAC contigs. However, even if the use of barley genomic resources circumvents the limitations caused by the complexity of the wheat genome, the divergence between the two species is large enough to observe synteny breaks. Thus, we performed a comparative study between wheat, barley and rice to assess the extent to which the barley gene order is transferable to wheat.

### Comparative genomics between wheat, barley and rice

In addition to its interest for anchoring physical maps, cross-species hybridisation also provides valuable data for comparative genomics as it allows the mapping of barley (and to some extent rice) orthologous genes on wheat chromosomes. We studied synteny, *i.e*. the conservation of the genes on the orthologous chromosomes of wheat chromosome 3B in barley (chromosome 3H) and rice (chromosome 1) without the assumption of the conservation of the gene order [42]. Out of the 738 probes located on the contigs of wheat chromosome 3B, 209 were mapped on barley chromosome 3 H and 99 on other barley chromosomes. Rice orthologous genes were identified unambiguously for 659 of the 738 probes located on wheat chromosome 3B, of which 389 are located on rice chromosome 1and 270 on other rice chromosomes (Table 1). These results suggest that, at the whole chromosome scale, 68% and 59% of wheat chromosome 3B genes are syntenic with genes located on the orthologous barley chromosome 3 H and rice chromosome 1, respectively. These results are consistent with previous studies that estimated between 59% and 74% of the genes were in conserved positions between wheat chromosome group 3 and rice chromosome 1 and 75% between wheat chromosome group 3 and barley chromosome 3 H [13,25,43-50]. The genes that are not syntenic between wheat chromosome 3B and barley chromosome 3 H mapped on the other barley chromosomes with no significant bias towards any other single chromosome (Chi^2 ^test, *P-*value = 0.16). In contrast, the non-syntenic genes between wheat chromosome 3B and rice chromosome 1 are biased in favour of genes mapped on the rice chromosomes carrying the highest number of genes (chromosomes 3 and 5) and against the rice chromosome carrying the lowest number of genes (chromosome 9) (Chi^2 ^test, *P-*value < 10^-5^). Further analyses confirmed that the distribution of the non-syntenic genes between wheat chromosome 3B and rice chromosome 1 on the other rice chromosomes is correlated with the number of genes per rice chromosome (Pearson's correlation coefficient r = 0.735; *P-*value = 0.01) [3]. Thus, no real mapping bias was identified towards any of the non-syntenic barley or rice chromosomes.

**Table 1:** Mapping data in wheat chromosome 3B deletion bins.

	Wheat			Barley			Rice		
**3B Deletion Bin**	**Bin size (Mb)**	**Number of loci**	**Density (locus/Mb)**	**Gene loci mapped on 3H**	**Gene loci mapped on the other chromosomes**	**Collinear gene loci between 3B and 3H**	**Gene loci mapped on Os01**	**Gene loci mapped on the other chromosomes**	**Collinear gene loci between 3B and Os01**

3BS8-0.78-1.00	44.2	37	0.84	10	9	7	15	14	10
3BS9-0.57-0.78	43.2	43	1.00	12	4	9	22	16	16
3BS1-0.33-0.57	94.3	86	0.91	14	11	8	40	33	20
C-3BS1-0.33	58.3	34	0.58	9	1	4	23	7	16
C-3BL2-0.22	45.7	36	0.79	15	3	8	27	6	18
3BL2-0.22-0.50	74.9	78	1.04	25	10	21	47	26	38
3BL10-0.50-0.63	40.1	40	1.00	13	5	8	18	17	8
3BL7-0.63-1.00	155.5	165	1.06	55	14	37	93	57	59
Total assigned	556.2	519	0.93	153	57	102	285	176	185
Not assigned	438.8	238	/	66	42	/	111	104	/
Total	995	757	/	219	99	/	396	280	/
				318			676		

Interestingly, a number of genes located on wheat chromosome 3B were not syntenic with barley chromosome 3 H but their homologs were syntenic between barley and rice. For example, 11 wheat chromosome 3B genes mapped on barley chromosome 2 H and on its ortholog in rice (chromosome 4). We found another example with 9 wheat chromosome 3B genes mapping on barley chromosome 6 H and on the orthologous rice chromosome 2 [44]. This result indicates that these genes have undergone rearrangements specifically in wheat and supports the recent finding of Choulet *et al*. [16] for extensive interchromosomal duplications in wheat.

Out of the 219 gene loci orthologous to barley chromosome 3 H genes, 153 have been located on wheat BACs assigned to one of the eight deletion bins of wheat chromosome 3B using the physical map data [17]. Their approximate location on the chromosome arms was thus inferred from the mapping data of the BAC. This enabled us to study the synteny between wheat, barley and rice at a finer scale. We calculated the percentage of probes that are syntenic to barley chromosome 3 H genes for each deletion bin of chromosome 3B and found that the conservation of genes is significantly uniform along chromosome 3B (Chi^2 ^test, *P-*value = 0.84) with 73% of syntenic genes per bin on average (Table 1). We performed the same calculation with the 285 genes assigned to wheat chromosome 3B deletion bins and syntenic to genes on rice chromosome 1. In this case, the distribution of syntenic genes was negatively correlated with the distance to the centromere (Pearson's correlation coefficient r = -0.742; *P-*value = 0.04). In other words, the level of synteny between wheat chromosome 3B and rice chromosome 1 decreases from the centromere to the telomeres. This is in complete agreement with the results of Akhunov *et al*. [39] who correlated this with the recombination rate along wheat chromosomes. However, using the data from Saintenac *et al*. [51] who performed an analysis of the distribution of the recombination rate among chromosome 3B, we did not find any correlation between the synteny level and crossing-over frequency (Pearson's correlation coefficient r = -0.378; *P-*value = 0.36). Comparisons between the sequences of 18 Mb sized contigs of chromosome 3B with the rice and *Brachypodium *genomes led to the same conclusions [16]. Moreover, the authors found a positive correlation between transposable element activity and the number of non syntenic genes. Thus, it is likely that the synteny level between wheat chromosome 3B and rice chromosome 1 that decreases from the centromere to the telomeres results from a combination of factors that have still to be identified.

**Figure 1:**
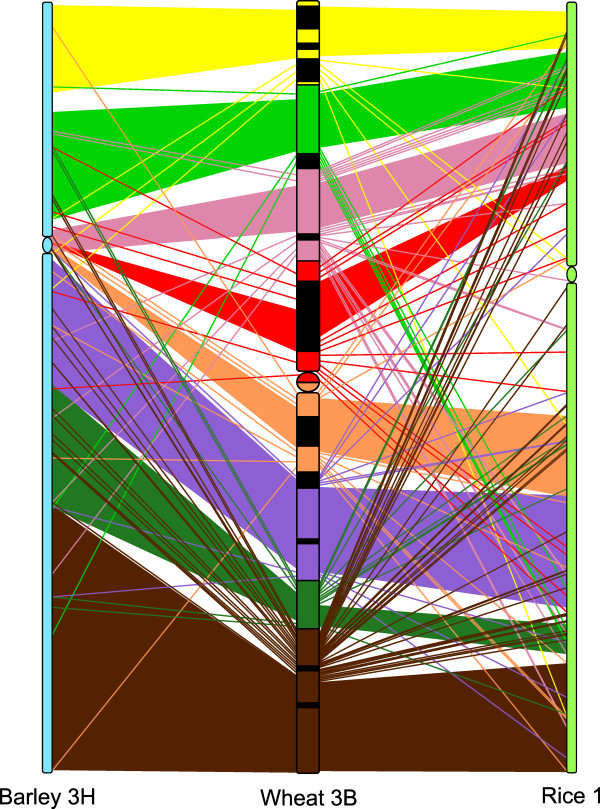
**Collinearity between wheat chromosome 3B, barley chromosome 3 H and rice chromosome 1**. Each colour on wheat chromosome 3B corresponds to a deletion bin. Yellow: 3BS8-0.78-1.00; light green: 3BS9-0.57-0.78; pink: 3BS1-0.33-0.57; red: C-3BS1-0.33; orange: C-3BL2-0.22; purple: 3BL2-0.22-0.50; dark green: 3BL10-0.50-0.63 and brown: 3BL7-0.63-1.00. The black segments correspond to the heterochromatic regions identified by C-banding, the coloured segments to the euchromatic regions and the circle to the centromere. The coloured blocks represent regions where the genes are collinear between two chromosomes. The lines represent the genes that are not collinear between two chromosomes. As the relative order of wheat genes in a given deletion bin is not known, we inferred this order from the barley chromosome 3 H and from rice chromosome 1 data.

The links between the barley chromosome 3 H genetic map, the rice chromosome 1 sequence and the wheat chromosome 3B deletion bin map were used to analyse the collinearity, *i.e*. the order of the genes [42], between the genes on wheat chromosome 3B and on barley chromosome 3 H and between the genes on wheat chromosome 3B and on rice chromosome 1. As the relative order of wheat genes in a given deletion bin is not known, we inferred this order from the barley chromosome 3 H and rice chromosome 1 data. This virtual order was used to define eight synteny blocks on barley chromosome 3 H and rice chromosome 1 corresponding to the eight deletion bins of wheat chromosome 3B (Figure 1). Genes being assigned to a given deletion bin of wheat and to the corresponding synteny block of barley or rice were considered as collinear. Those not being conserved at syntenic positions were considered as non collinear. In total we found that 102 (67%) genes were collinear between wheat 3B and barley 3 H and 185 (65%) between wheat 3B and rice 1 (Table 1 & Figure 1). We calculated the percentage of collinear genes between wheat chromosome 3B and barley chromosome 3 H and between wheat chromosome 3B and rice chromosome 1 for each bin. We found that the distribution of collinear genes is significantly uniform along chromosome 3B (Chi^2 ^test, *P-*value = 0.89 and 0.69 for barley and rice respectively). Some translocations of genes can be observed between wheat chromosome 3B and barley chromosome 3 H and between wheat chromosome 3B and rice chromosome 1 (Figure 1) that are similar to previous studies [13,52]. However, we expected a higher collinearity between the wheat and barley group 3 chromosomes with regard to previous results [29-32]. We think that this apparent discrepancy may originate from the construction of the barley neighbour map. None of the five individual barley genetic maps available to date holds a sufficient number of genes, and therefore we had to use a barley neighbour map combining these maps to optimize the anchoring experiment. However, the gene order is not fully reliable in such maps especially in the pericentromeric and centromeric parts of the chromosomes where recombination is reduced or totally suppressed [25]. Finally, the use of wheat neighbour genetic mapping data suggests additional rearrangements in bins (data not shown) as suggested by Liu *et al*. [53] and Chantret *et al*. [54] and this may also lead to some discrepancies.

Altogether, our results regarding conservation between wheat chromosome 3B, barley chromosome 3 H and rice chromosome 1 at the whole chromosome and at the deletion bins scales are in agreement with previous studies. However, we also noticed some wheat-specific rearrangements of the genes that disrupt the collinearity between wheat and barley and between wheat and rice. Thus, globally we expected the genes to be in the same order between wheat and barley but rearrangements are likely to be observed locally. So the results of anchoring and ordering of the wheat BAC contigs along chromosome 3B using the barley mapping data should be considered with caution as they may not be perfectly exact.

### Wheat gene space organisation

**Figure 2:**
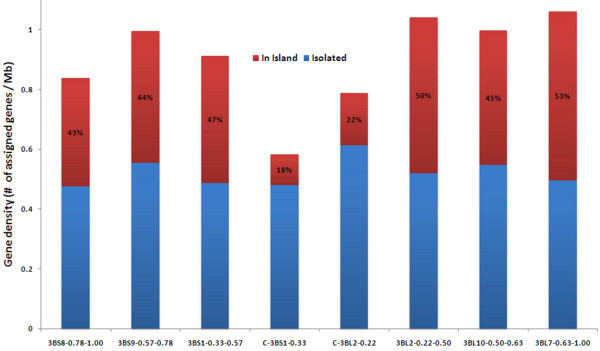
**Gene density in the eight deletion bins of wheat chromosome 3B**. The density of isolated genes is represented by blue bars. The density of the genes organized in island is represented by red bars. The proportions of genes organized in island per deletion bins are shown as percentages within the red bars.

For the first time, we were able to assign precisely a large number of genes to individual BACs and BAC contigs whose order is known on wheat chromosome 3B. This led us to analyse the pattern of gene distribution along the chromosome. Out of the 757 loci mapped on the wheat chromosome 3B physical map, 519 loci were assigned to the eight deletion bins (Table 1). The density of loci per deletion bin was calculated by dividing the number of loci assigned to each deletion bin by the cumulative length of contigs in the bin [17]. The density of loci showed a slight increasing gradient from the centromere to the telomeres (Figure 2) with a positive correlation with the distance to the centromere (Pearson's correlation coefficient r = 0.664), even though this correlation was not statistically significant using a 5% threshold (*P-*value = 0.07). The highest density was found on the most distal 3BL7-0.63-1.00 deletion bin of the long arm (1.06 probes per megabase), whereas the lowest density was observed on the most proximal C-3BS1-0.33 bin of the short arm (0.58 probes per megabase). Such a gradient could be artificially created by a difference in gene sequence conservation from the centromere to the telomeres between wheat and barley. To test this hypothesis, we calculated the coefficient of correlation between the gene density and the percentage of sequence identity per bin. No significant correlation was found (Pearson's correlation coefficient r = -0.478; *P-*value = 0.23), demonstrating that the gene density gradient is not biased by the differences in the similarity between wheat and barley gene sequences. Thus, the gene density distribution observed here provides a reliable snapshot of the gene space organisation along wheat chromosome 3B and led to a hypothesis where the gene density is higher in the distal parts than in the proximal parts of the chromosome (Figure 2). As we found that 87% of the genes were mapped on the 81% most distal parts of chromosome 3B, our result is in agreement with the moderate gradient of gene density along wheat chromosomes suggested by Munkvold *et al*. [13], Devos *et al*. [15], Charles *et al*. [14] and more recently, Choulet *et al*. [16]. The discrepancy between these results and the first suggestions of Erayman *et al*. [12] that most of the genes are located in the distal parts of the wheat chromosomes may originate from their use of a consensus deletion bin map from the A, B and D chromosomes, where markers were non-systematically assigned to the deletion bins [51]. This likely led to approximations of the relative positions of the markers and the postulation that genes are mostly found in gene-rich regions in wheat. Here, the possibility to assign genes on physical contigs that cover 82% of a specific chromosome 3B [17] enabled us to derive more precise information about the gene distribution.

We then extrapolated the expected gene density per deletion bin to the whole set of chromosome 3B genes. We first estimated the number of genes per bin by considering the bins fully covered by contigs and by keeping the same gene density distribution along chromosome 3B. This resulted in an estimate of 904 loci assigned to the eight deletion bins compared to the 519 loci identified by hybridisation in this study. Recently Choulet *et al*. [16] estimated that chromosome 3B carries 8400 genes. We then extrapolated the gene density by considering 8400 loci assigned to the eight deletion bins. We found that the distal bin 3BL7-0.63-1.00 and the proximal bin C-3BS1-0.33 would have a gene density of 1 gene per 101 kb and 1 per 185 kb, respectively. Therefore, even in the least gene-dense regions of the chromosome, our results indicate that there may be one gene on average every 185 kb and therefore no megabase-sized regions devoid of genes. This is consistent with RNA hybridisations on chromosome 3B MTP arrays that showed that the largest region without genes is about 800 kb long and that genes are distributed across the entire chromosome 3B [16].

However, our approach suffers from a major limitation to estimate the gene density precisely. Here, the gene densities estimated for the 3BS8-0.78-1.00 and 3BL7-0.63-1.00 telomeric deletion bins were lower than the ones found through RFLP hybridisation with ESTs by Munkvold *et al*. [13] (normalized gene densities: 0.928 *versus *1.190 and 1.176 *versus *1.421, respectively). One of the characteristics of telomeric parts of wheat chromosomes is that they accumulate tandemly duplicated genes at a high rate [16,39]. Thus, it is likely that the differences in gene density observed between the two experiments reflect the inability of gene mapping based on BAC hybridisation to detect tandemly duplicated genes. This method is only qualitative and detects the presence or absence of a gene on a BAC but it does not indicate whether a gene located on a BAC is present in single or multiple copies. Thus, the gene density established through gene mapping based on BAC hybridisation is likely underestimated in the distal regions and therefore one can expect an even higher gene density gradient. If we consider that the difference in gene density between the two studies is only due to tandemly duplicated genes, we could estimate that we missed 28% and 21% of genes for 3BS8-0.78-1.00 and 3BL7-0.63-1.00 deletion bins, respectively. This also explains why our estimation of gene density at telomeres was lower compared to the gene density of sequenced contigs located in distal regions of chromosome 3B (1 gene per 101 kb *versus *1 gene per 86 kb) whereas our estimation at the centromere precisely fits the gene density of sequenced contigs located in proximal regions (1 gene per 185 kb *versus *1 gene per 184 kb). Assuming that we missed 21% of genes due to tandem duplications in 3BL7-0.63-1.00 deletion bin, the gene density would be 1 gene per 90 kb. This demonstrates that all these studies can give an indication of the general gene space organisation along wheat chromosomes but are unable to precisely estimate the local gene density of specific regions.

To further study the gene space and especially the genes clustered in islands in more detail, we considered gene islands as multiple genes located on the same BAC or overlapping BACs, *i.e*. separated by less than 150 kb. Out of the 757 loci mapped on wheat chromosome 3B physical map, 303 loci, *i.e*. 40%, were considered part of gene islands, whereas the 454 remaining genes (60%) were considered as isolated genes. In contrast to the distribution of isolated genes that we found significantly uniform along chromosome 3B (Chi^2 ^test, *P-*value = 0.97), the distribution of genes organised in islands was significantly non-uniform along chromosome 3B (Chi^2 ^test, *P-*value < 10^-5^) with a positive correlation between the density of genes in islands and the distance to the centromere (Pearson's correlation coefficient r = 0.762; *P-*value = 0.03) (Figure 2). We also found a correlation between the density of genes in islands and the overall gene density (Pearson's correlation coefficient r = 0.956, *P-*value < 10^-3^). This strongly suggests that the gradient of gene density between centromeric and telomeric regions is due to the differential distribution of genes organised in islands across the chromosome with proportionately more genes in islands in the distal parts compared to the proximal parts.

In conclusion, our cross-species hybridisation technique allowed us to assign a large number of genes onto wheat chromosome 3B at the BAC resolution and to obtain original results on the wheat gene space organisation. We confirmed that the gene density distribution along the chromosome 3B follows a slight gradient from the centromere to the telomeres and we suggest that the presence of more gene islands in the distal part of the chromosome explains this gradient. However, the ultimate experiment to access the whole set of genes and confirm the gene density distribution at a high resolution along a wheat chromosome will be high-quality sequencing and annotation. This is currently underway for chromosome 3B (C. Feuillet, personal communication).

## Conclusions

Our study demonstrates that hybridisations of the barley Agilent 15K expression microarray with wheat chromosome 3B MTP pools is a convenient and cost-efficient technique to perform physical map anchoring with gene-based markers. Our comparative genomics analysis between wheat, barley and rice confirms good global collinearity between these species, with a few wheat-specific rearrangements that could lead to local mis-ordering of wheat contigs using the barley gene order. Using this technique, we also confirmed previous studies that the gene space organisation follows a gradient of gene density along chromosome 3B from centromere to telomeres without large "gene-free" regions. We also demonstrated that this gradient was generated by a differential accumulation of gene islands between the centromere and the telomeres with more genes in islands in the distal parts of the chromosome. Such results have far-reaching implications in terms of strategies to sequence the wheat genome. Indeed, our results confirm that to access the whole wheat gene set, the entire wheat genome needs to be sequenced. A wheat expression microarray is currently being utilised to increase the density of genes at the BAC scale located along wheat chromosome 3B and to improve our understanding of the wheat gene space organisation.

## Methods

### Barley expression microarray and hybridisations

The barley Agilent 15K expression microarray contains 15208 barley 60-mer probes derived from unigenes of HarvEST assembly #25 used to originally design probe sets for the 22K Barley1 Affymetrix GeneChip [21]. BACs (7440 in total) arranged in twenty 384-well plates were selected to build a wheat chromosome 3B Minimal Tiling Path covering 82% of the whole chromosome with ~30% overlap as described by Paux *et al*. [17]. These twenty plates were pooled in three dimensions (20 plate pools, 16 row pools and 24 column pools) to generate 60 samples by CNRGV (Toulouse, France) and the BAC pools were amplified as described by Paux *et al*. [17]. Two channels processing of the microarrays was used, with BAC pool DNA labelled with Cy3 and a mixed reference set of barley cv. Golden Promise RNAs (equal amounts of leaf, root and inflorescence) labelled with Cy5. RNA (5 μg) was labelled as described by Ducreux *et al*. [55]. Amplified BAC pool DNA (200 ng) was labelled using a modified BioPrime Genomic DNA Labelling System (Invitrogen, Carlsbad, California USA): BAC pool DNA in 11 μl was added to 10 μl Random Primer Reaction Buffer mix and denatured at 95°C for 5 min prior to cooling on ice and to this was added 2.5 μl modified 10× dNTPs buffer (1.2 mM each of dATP, dGTP, dTTP; 0.6 mM dCTP; 10 mM Tris pH8.0; 1 mM EDTA), Cy3 dCTP (1 μl of 1 nM) and 0.5 μl Klenow enzyme (20U) followed by incubation for 16 h at 37°C. Labelled samples (BAC DNA & reference RNA) for each array were combined and unincorporated dyes removed using the Qiaquick PCR Purification Kit (Qiagen, Hilden, Germany) as recommended, eluting with 20 μl EB buffer (Qiagen, Hilden, Germany). Hybridisations and washing were carried out as recommended (Agilent Protocol v5.5). Scanning was performed with an Agilent G2505B scanner using default settings and data extracted using Agilent FE software (v 9.5.3). All data has been submitted to ArrayExpress [56] (accession # E-TABM-1011) under MIAME guidelines [57].

### Blast analyses

A BLASTN analysis [33] was performed with the 60-mer barley probes against the TREP database [34] to identify probes that could hybridise with TEs of the wheat BACs. We considered that a probe could generate a false positive due to TEs if we found 80% identity on a minimum 45 nucleotides. Then a BLASTN analysis [33] was performed with the 15208 60-mer barley probes against the sequenced contigs of wheat chromosome 3B [16]. The annotation of the best hit on the sequenced contigs was viewed using Artemis [58]. The best hit and the query barley probe were then aligned using ClustalW2 [59] and the sequence identity was calculated using the entire barley probe length. In addition, a BLASTN analysis [33] was performed with the 60-mer barley probes against 6162 wheat *cv *Chinese Spring full-length cDNAs developed by Kawaura *et al*. [38]. The sequence identity between the best hit and the query barley probe was calculated on the entire barley probe length as previously described. The most significant rice homologues to the unigenes used to design the barley microarray probes were identified by BLASTN searches of the gene models from the Rice Genome Annotation Project from Michigan State University (Rice Pseudomolecules v5 database [60]).

### Data deconvolution

Following hybridisation, signals were analysed to rebuild the MTP addresses of the BACs carrying an ortholog of a barley probe. For each barley probe, we identified the positive pools to determine the original MTP BAC address on which it is located. Each type of pool does not contain the same number of BACs (plate: 384 BACs/pool; row: 480 BACs/pool; column: 320 BACs/pool).

The first normalisation step undertaken addressed the fact that the 60 samples had different hybridisation signal averages. Medians were calculated for each pool independently and each value was divided by the median corresponding to the pool type. This led to comparable hybridisation values for each pool. A second normalisation step was undertaken for each probe, based on the same method. After this second normalisation step, probe hybridisation values were all comparable, while pool hybridisation values were not significantly changed.

To identify pools with positive signal, we first used an automated classical outlier detection method, that we called the "automated scoring" method. The mean and the standard deviation were calculated for each probe and used to define a different threshold for each probe. Calculation of the thresholds was different for each pool type (plate: Mean + 2.8 × Standard Deviation; row: Mean + 2.5 × Standard Deviation; column: Mean + 3 × Standard Deviation). All the pools with probe signal above this threshold were considered positive. We repeated this step twice by deleting positive signals previously detected, calculating the mean, standard deviation and the threshold again for each probe and selecting the new positive signals above the new thresholds. The calculation of the thresholds for the three pool types remained the same.

Following this "automated scoring" method, a "semi-automated" one was performed to identify missing coordinates from probes having five positive pools (*e.g*. two plate coordinates, two row coordinates and one column coordinate). Here, a combination of all possible coordinates was used to try to identify two overlapping BACs. A "manual scoring" analysis was also performed to identify the missing coordinate from probes having two positive pools.

The final analysis that we called "boxplot scoring" method was performed whereby a boxplot was drawn using R software [61] for each probe for each of the three pool types and the upper outlier values were considered as positive pools. This analysis was less stringent than the "automated scoring" so we kept only the probes that were located on two overlapping BACs.

To rebuild the BAC addresses, we collated the positive pools for each probe. Some probes gave one positive pool per pool type which enables us to identify an unambiguous BAC address. However, some probes gave more positive pools per pool type. We therefore looked at every combination and used the chromosome 3B physical map data [17] to assist finding addresses of overlapping BACs where the probe is located on the overlap itself.

After identification of BACs carrying barley orthologs, we used the physical map [17] to locate them in their respective contig and possibly in one of the eight chromosome 3B deletion bins used for this study.

### Synteny and collinearity analyses

In total, five barley genetic maps [21-25] were used to establish a barley neighbour map using the same criteria as the IBM neighbour map of maize [41]. For two maps [24,25], we had to perform a BLASTN analysis to link the markers to the barley unigenes mapped on wheat chromosome 3B. The best hits with at least 85% identity over 100 nucleotides were selected for each unigene. The order of the rice genes along the chromosomes was established using the rice gene numbering annotation. As the relative order of wheat genes in a given deletion bin is not known, we inferred this order from the barley chromosome 3 H and rice chromosome 1 data. The software GenomePixelizer [62] was used for the graphical display of the collinearity between wheat chromosome 3B, barley chromosome 3 H and rice chromosome 1.

### Statistical analyses

The statistical analyses including the T-test, Chi^2 ^and Pearson's correlation coefficient tests were performed using R software [61] at a 5% threshold. The distance to centromere was estimated from the centromere to the middle of deletion bins. We calculated the gene density per deletion bins by dividing the number of genes assigned to the bin by the length of the contigs assigned to the same bin. The gene density per bin of Munkvold *et al*. [13] was estimated by dividing the number of genes they assigned to the bin by the total size of the bin. The normalized gene densities per deletion bin were calculated by dividing the density for each bin by the mean of the densities along chromosome 3B. The Rice Genome Annotation Project from Michigan State University (Rice Pseudomolecules v6.1 database [60]) was used to estimate the number of annotated genes on the rice chromosomes. For the Chi^2 ^tests, to test the uniformity of the percentages and the densities along chromosome 3B, we estimated the number of genes per deletion bins that would have generated a uniform distribution and we used these numbers as theoretical values.

## Abbreviations

MTP: minimal tiling path; TE: transposable element.

## Authors' contributions

PH and JM carried out the hybridisations; CR, PH and FC performed data analysis; RW, CF and EP helped with the interpretation of the results; CR drafted the manuscript; FC, RW, CF and EP were involved in improving the manuscript. The final version of the manuscript was approved by all the authors.

## References

[B1] Jackson S Hass Jacobus B Pagel J Wilson RF, Stalker HT, Brummer EC The Gene Space of the Soybean Genome Legume Crop Genomics 2004 Champaign: AOCS Press 187 193

[B2] Varshney RK Hoisington DA Tyagi AK Advances in cereal genomics and applications in crop breeding Trends Biotechnol 2006 24 490 499 10.1016/j.tibtech.2006.08.006 16956681

[B3] International Rice Genome Sequencing Project The map-based sequence of the rice genome Nature 2005 436 793 800 10.1038/nature03895 16100779

[B4] The Arabidopsis Genome Initiative Analysis of the genome sequence of the flowering plant Arabidopsis thaliana Nature 2000 408 796 814 10.1038/35048692 11130711

[B5] The International Brachypodium Initiative Genome sequencing and analysis of the model grass Brachypodium distachyon Nature 2010 463 763 768 10.1038/nature08747 20148030

[B6] Jaillon O Aury JM Noel B Policriti A Clepet C Casagrande A Choisne N Aubourg S Vitulo N Jubin C Vezzi A Legeai F Hugueney P Dasilva C Horner D Mica E Jublot D Poulain J Bruyere C Billault A Segurens B Gouyvenoux M Ugarte E Cattonaro F Anthouard V Vico V Del Fabbro C Alaux M Di Gaspero G Dumas V The grapevine genome sequence suggests ancestral hexaploidization in major angiosperm phyla Nature 2007 449 463 467 10.1038/nature06148 17721507

[B7] Tuskan GA Difazio S Jansson S Bohlmann J Grigoriev I Hellsten U Putnam N Ralph S Rombauts S Salamov A Schein J Sterck L Aerts A Bhalerao RR Bhalerao RP Blaudez D Boerjan W Brun A Brunner A Busov V Campbell M Carlson J Chalot M Chapman J Chen GL Cooper D Coutinho PM Couturier J Covert S Cronk Q The genome of black cottonwood, Populus trichocarpa (Torr. & Gray) Science 2006 313 1596 1604 10.1126/science.1128691 16973872

[B8] Schmutz J Cannon SB Schlueter J Ma J Mitros T Nelson W Hyten DL Song Q Thelen JJ Cheng J Xu D Hellsten U May GD Yu Y Sakurai T Umezawa T Bhattacharyya MK Sandhu D Valliyodan B Lindquist E Peto M Grant D Shu S Goodstein D Barry K Futrell-Griggs M Abernathy B Du J Tian Z Zhu L Genome sequence of the palaeopolyploid soybean Nature 2010 463 178 183 10.1038/nature08670 20075913

[B9] Schnable PS Ware D Fulton RS Stein JC Wei F Pasternak S Liang C Zhang J Fulton L Graves TA Minx P Reily AD Courtney L Kruchowski SS Tomlinson C Strong C Delehaunty K Fronick C Courtney B Rock SM Belter E Du F Kim K Abbott RM Cotton M Levy A Marchetto P Ochoa K Jackson SM Gillam B The B73 maize genome: complexity, diversity, and dynamics Science 2009 326 1112 1115 10.1126/science.1178534 19965430

[B10] Zonneveld BJ Leitch IJ Bennett MD First nuclear DNA amounts in more than 300 angiosperms Ann Bot (Lond) 2005 96 229 244 10.1093/aob/mci170 PMC4246870 15905300

[B11] Endo TR Gill BS The deletion stocks of common wheat J Hered 1996 87 295 307

[B12] Erayman M Sandhu D Sidhu D Dilbirligi M Baenziger PS Gill KS Demarcating the gene-rich regions of the wheat genome Nucleic Acids Res 2004 32 3546 3565 10.1093/nar/gkh639 15240829 PMC484162

[B13] Munkvold JD Greene RA Bermudez-Kandianis CE La Rota CM Edwards H Sorrells SF Dake T Benscher D Kantety R Linkiewicz AM Dubcovsky J Akhunov ED Dvorak J Gustafson JP Pathan MS Nguyen HT Matthews DE Chao S Lazo GR Hummel DD Anderson OD Anderson JA Gonzalez-Hernandez JL Peng JH Lapitan N Qi LL Echalier B Gill BS Hossain KG Group 3 Chromosome Bin Maps of Wheat and Their Relationship to Rice Chromosome 1 Genetics 2004 168 639 650 10.1534/genetics.104.034819 15514041 PMC1448823

[B14] Charles M Belcram H Just J Huneau C Viollet A Couloux A Segurens B Carter M Huteau V Coriton O Appels R Samain S Chalhoub B Dynamics and differential proliferation of transposable elements during the evolution of the B and A genomes of wheat Genetics 2008 180 1071 1086 10.1534/genetics.108.092304 18780739 PMC2567357

[B15] Devos KM Ma J Pontaroli AC Pratt LH Bennetzen JL Analysis and mapping of randomly chosen bacterial artificial chromosome clones from hexaploid bread wheat Proc Natl Acad Sci USA 2005 102 19243 19248 10.1073/pnas.0509473102 16357197 PMC1323192

[B16] Choulet F Wicker T Rustenholz C Paux E Salse J Leroy P Schlub S Le Paslier MC Magdelenat G Gonthier C Couloux A Budak H Breen J Pumphrey M Liu S Kong X Jia J Gut M Brunel D Anderson JA Gill BS Appels R Keller B Feuillet C Megabase Level Sequencing Reveals Contrasted Organization and Evolution Patterns of the Wheat Gene and Transposable Element Spaces Plant Cell 2010 22 1686 1701 10.1105/tpc.110.074187 20581307 PMC2910976

[B17] Paux E Sourdille P Salse J Saintenac C Choulet F Leroy P Korol A Michalak M Kianian S Spielmeyer W Lagudah E Somers D Kilian A Alaux M Vautrin S Bergès H Eversole K Appels R Safar J Simkova H Dolezel J Bernard M Feuillet C A physical map of the 1Gb bread wheat chromosome 3B Science 2008 322 101 104 10.1126/science.1161847 18832645

[B18] Lehmensiek A Bovill W Wenzl P Langridge P Appels R Feuillet C, Muelhlbauer G Genetic Mapping in the Triticeae Genetics and Genomics of the Triticeae 2009 Berlin: Springer 201 235 full_text

[B19] Paux E Sourdille P Feuillet C, Muelhlbauer G A Toolbox for Triticeae Genomics Genetics and Genomics of the Triticeae 2009 Berlin: Springer 255 283 full_text

[B20] GrainGenes 2.0 http://wheat.pw.usda.gov/GG2

[B21] Chen X Hackett CA Niks RE Hedley PE Booth C Druka A Marcel TC Vels A Bayer M Milne I Morris J Ramsay L Marshall D Cardle L Waugh R An eQTL analysis of partial resistance to Puccinia hordei in barley PLoS One 2010 5 e8598 10.1371/journal.pone.0008598 20066049 PMC2798965

[B22] Close TJ Bhat PR Lonardi S Wu Y Rostoks N Ramsay L Druka A Stein N Svensson JT Wanamaker S Bozdag S Roose ML Moscou MJ Chao S Varshney RK Szucs P Sato K Hayes PM Matthews DE Kleinhofs A Muehlbauer GJ DeYoung J Marshall DF Madishetty K Fenton RD Condamine P Graner A Waugh R Development and implementation of high-throughput SNP genotyping in barley BMC Genomics 2009 10 582 10.1186/1471-2164-10-582 19961604 PMC2797026

[B23] Potokina E Druka A Luo Z Wise R Waugh R Kearsey M Gene expression quantitative trait locus analysis of 16000 barley genes reveals a complex pattern of genome-wide transcriptional regulation The Plant Journal 2008 53 90 101 10.1111/j.1365-313X.2007.03315.x 17944808

[B24] Sato K Nankaku N Takeda K A high-density transcript linkage map of barley derived from a single population Heredity 2009 103 110 117 10.1038/hdy.2009.57 19455180

[B25] Stein N Prasad M Scholz U Thiel T Zhang HN Wolf M Kota R Varshney RK Perovic D Grosse I Graner A A 1,000-loci transcript map of the barley genome: new anchoring points for integrative grass genomics Theor Appl Genet 2007 114 823 839 10.1007/s00122-006-0480-2 17219208

[B26] Chalupska D Lee HY Faris JD Evrard A Chalhoub B Haselkorn R Gornicki P Acc homoeoloci and the evolution of wheat genomes Proc Natl Acad Sci USA 2008 105 9691 9696 10.1073/pnas.0803981105 18599450 PMC2474508

[B27] Dvorak J Akhunov ED Tempos of gene locus deletions and duplications and their relationship to recombination rate during diploid and polyploid evolution in the Aegilops-Triticum alliance Genetics 2005 171 323 332 10.1534/genetics.105.041632 15996988 PMC1456522

[B28] Wicker T Narechania A Sabot F Stein J Vu GT Graner A Ware D Stein N Low-pass shotgun sequencing of the barley genome facilitates rapid identification of genes, conserved non-coding sequences and novel repeats BMC Genomics 2008 9 518 10.1186/1471-2164-9-518 18976483 PMC2584661

[B29] Bennetzen JL Ramakrishna W Numerous small rearrangements of gene content, order and orientation differentiate grass genomes Plant Mol Biol 2002 48 821 827 10.1023/A:1014841515249 11999852

[B30] Devos KM Gale MD Genome relationships: The grass model in current research Plant Cell 2000 12 637 646 10.1105/tpc.12.5.637 10810140 PMC139917

[B31] Dubcovsky J Luo MC Zhong GY Bransteitter R Desai A Kilian A Kleinhofs A Dvorak J Genetic map of diploid wheat, Triticum monococcum L, and its comparison with maps of Hordeum vulgare L Genetics 1996 143 983 999 8725244 10.1093/genetics/143.2.983 PMC1207354

[B32] Moore G Devos KM Wang Z Gale MD Cereal genome evolution. Grasses, line up and form a circle Curr Biol 1995 5 737 739 10.1016/S0960-9822(95)00148-5 7583118

[B33] Altschul SF Madden TL Schaffer AA Zhang JH Zhang Z Miller W Lipman DJ Gapped BLAST and PSI-BLAST: a new generation of protein database search programs Nucleic Acids Res 1997 25 3389 3402 10.1093/nar/25.17.3389 9254694 PMC146917

[B34] Wicker T Matthews DE Keller B TREP: a database for Triticeae repetitive elements Trends Plant Sci 2002 7 561 562 10.1016/S1360-1385(02)02372-5

[B35] Mayer KF Taudien S Martis M Simkova H Suchankova P Gundlach H Wicker T Petzold A Felder M Steuernagel B Scholz U Graner A Platzer M Dolezel J Stein N Gene content and virtual gene order of barley chromosome 1H Plant Physiol 2009 151 496 505 10.1104/pp.109.142612 19692534 PMC2754631

[B36] Suchánková P Kubaláková M KováŐová P Bartoš J Číhalíková J Molnár-Láng M Endo T Doležel J Dissection of the nuclear genome of barley by chromosome flow sorting Theor Appl Genet 2006 113 651 659 16810504 10.1007/s00122-006-0329-8

[B37] Letowski J Brousseau R Masson L Designing better probes: effect of probe size, mismatch position and number on hybridization in DNA oligonucleotide microarrays J Microbiol Methods 2004 57 269 278 10.1016/j.mimet.2004.02.002 15063067

[B38] Kawaura K Mochida K Enju A Totoki Y Toyoda A Sakaki Y Kai C Kawai J Hayashizaki Y Seki M Shinozaki K Ogihara Y Assessment of adaptive evolution between wheat and rice as deduced from full-length common wheat cDNA sequence data and expression patterns BMC Genomics 2009 10 271 10.1186/1471-2164-10-271 19534823 PMC2703658

[B39] Akhunov ED Goodyear AW Geng S Qi LL Echalier B Gill BS Gustafson JP Lazo G Chao S Anderson OD Linkiewicz AM Dubcovsky J La Rota M Sorrells ME Zhang D Nguyen HT Kalavacharla V Hossain K Kianian SF Peng J Lapitan NL Gonzalez-Hernandez JL Anderson JA Choi DW Close TJ Dilbirligi M Gill KS Walker-Simmons MK Steber C The organization and rate of evolution of wheat genomes are correlated with recombination rates along chromosome arms Genome Res 2003 13 753 763 10.1101/gr.808603 12695326 PMC430889

[B40] Klein PE Klein RR Cartinhour SW Ulanch PE Dong J Obert JA Morishige DT Schlueter SD Childs KL Ale M Mullet JE A high-throughput AFLP-based method for constructing integrated genetic and physical maps: progress toward a sorghum genome map Genome Res 2000 10 789 807 10.1101/gr.10.6.789 10854411 PMC310885

[B41] Cone KC McMullen MD Bi IV Davis GL Yim YS Gardiner JM Polacco ML Sanchez-Villeda H Fang Z Schroeder SG Havermann SA Bowers JE Paterson AH Soderlund CA Engler FW Wing RA Coe EH Jr Genetic, physical, and informatics resources for maize. On the road to an integrated map Plant Physiol 2002 130 1598 1605 10.1104/pp.012245 12481043 PMC1540265

[B42] Keller B Feuillet C Colinearity and gene density in grass genomes Trends Plant Sci 2000 5 246 251 10.1016/S1360-1385(00)01629-0 10838615

[B43] Bilgic H Cho S Garvin DF Muehlbauer GJ Mapping barley genes to chromosome arms by transcript profiling of wheat-barley ditelosomic chromosome addition lines Genome 2007 50 898 906 10.1139/G07-059 18059553

[B44] Bolot S Abrouk M Masood-Quraishi U Stein N Messing J Feuillet C Salse J The 'inner circle' of the cereal genomes Curr Opin Plant Biol 2009 12 119 125 10.1016/j.pbi.2008.10.011 19095493

[B45] Cho S Garvin DF Muehlbauer GJ Transcriptome analysis and physical mapping of barley genes in wheat-barley chromosome addition lines Genetics 2006 172 1277 1285 10.1534/genetics.105.049908 16322516 PMC1456225

[B46] Devos KM Updating the 'crop circle' Curr Opin Plant Biol 2005 8 155 162 10.1016/j.pbi.2005.01.005 15752995

[B47] Gaut BS Evolutionnary dynamics of grass genomes New Phytol 2002 154 15 28 10.1046/j.1469-8137.2002.00352.x

[B48] La Rota M Sorrells ME Comparative DNA sequence analysis of mapped wheat ESTs reveals the complexity of genome relationships between rice and wheat Funct Integr Genomics 2004 4 34 46 10.1007/s10142-003-0098-2 14740255

[B49] Thiel T Graner A Waugh R Grosse I Close TJ Stein N Evidence and evolutionary analysis of ancient whole-genome duplication in barley predating the divergence from rice BMC Evol Biol 2009 9 209 10.1186/1471-2148-9-209 19698139 PMC2746218

[B50] Varshney RK Sigmund R Börner A Korzun V Stein N Sorrells ME Langridge P Graner A Interspecific transferability and comparative mapping of barley EST-SSR markers in wheat, rye and rice Plant Science 2005 168 195 202 10.1016/j.plantsci.2004.08.001

[B51] Saintenac C Falque M Martin OC Paux E Feuillet C Sourdille P Detailed Recombination Studies along Chromosome 3B Provide New Insights on Crossover Distribution in Wheat (*Triticum aestivum *L) Genetics 2009 181 393 403 10.1534/genetics.108.097469 19064706 PMC2644935

[B52] Salse J Bolot S Throude M Jouffe V Piegu B Quraishi UM Calcagno T Cooke R Delseny M Feuillet C Identification and characterization of shared duplications between rice and wheat provide new insight into grass genome evolution Plant Cell 2008 20 11 24 10.1105/tpc.107.056309 18178768 PMC2254919

[B53] Liu S Zhang X Pumphrey MO Stack RW Gill BS Anderson JA Complex microcolinearity among wheat, rice, and barley revealed by fine mapping of the genomic region harboring a major QTL for resistance to Fusarium head blight in wheat Funct Integr Genomics 2005 1 7 10.1007/s10142-005-0007-y 16270217

[B54] Chantret N Salse J Sabot F Bellec A Laubin B Dubois I Dossat C Sourdille P Joudrier P Gautier MF Cattolico L Beckert M Aubourg S Weissenbach J Caboche M Leroy P Bernard M Chalhoub B Contrasted microcolinearity and gene evolution within a homoeologous region of wheat and barley species J Mol Evol 2008 66 138 150 10.1007/s00239-008-9066-8 18274696

[B55] Ducreux LJ Morris WL Prosser IM Morris JA Beale MH Wright F Shepherd T Bryan GJ Hedley PE Taylor MA Expression profiling of potato germplasm differentiated in quality traits leads to the identification of candidate flavour and texture genes J Exp Bot 2008 59 4219 4231 10.1093/jxb/ern264 18987392 PMC2639024

[B56] ArrayExpress http://www.ebi.ac.uk/microarray-as/ae/

[B57] Minimum Information About a Microarray Experiment - MIAME http://www.mged.org/Workgroups/MIAME/miame.html

[B58] Rutherford K Parkhill J Crook J Horsnell T Rice P Rajandream MA Barrell B Artemis: sequence visualization and annotation Bioinformatics 2000 16 944 945 10.1093/bioinformatics/16.10.944 11120685

[B59] Larkin MA Blackshields G Brown NP Chenna R McGettigan PA McWilliam H Valentin F Wallace IM Wilm A Lopez R Thompson JD Gibson TJ Higgins DG Clustal W and Clustal × version 2.0 Bioinformatics 2007 23 2947 2948 10.1093/bioinformatics/btm404 17846036

[B60] Rice Genome Annotation from Michigan State University http://rice.plantbiology.msu.edu/

[B61] R software http://www.r-project.org

[B62] GenomePixelizer http://www.atgc.org/GenomePixelizer/GenomePixelizer_Welcome.html

